# Integrating fish models in tuberculosis vaccine development

**DOI:** 10.1242/dmm.045716

**Published:** 2020-08-24

**Authors:** Anni K. Saralahti, Meri I. E. Uusi-Mäkelä, Mirja T. Niskanen, Mika Rämet

**Affiliations:** 1 Laboratory of Experimental Immunology, BioMediTech, Faculty of Medicine and Health Technology, Tampere University, Tampere FI-33014, Finland; 2 Vaccine Research Center, Faculty of Medicine and Health Technology, Tampere University, Tampere FI-33014, Finland; 3 PEDEGO Research Unit, Medical Research Center, University of Oulu, Oulu FI-90014, Finland; 4 Department of Children and Adolescents, Oulu University Hospital, Oulu FI-90029, Finland

**Keywords:** Tuberculosis vaccine, Zebrafish, Animal models, Mycobacteria

## Abstract

Tuberculosis is a chronic infection by *Mycobacterium tuberculosis* that results in over 1.5 million deaths worldwide each year. Currently, there is only one vaccine against tuberculosis, the Bacillus Calmette–Guérin (BCG) vaccine. Despite widespread vaccination programmes, over 10 million new *M. tuberculosis* infections are diagnosed yearly, with almost half a million cases caused by antibiotic-resistant strains. Novel vaccination strategies concentrate mainly on replacing BCG or boosting its efficacy and depend on animal models that accurately recapitulate the human disease. However, efforts to produce new vaccines against an *M. tuberculosis* infection have encountered several challenges, including the complexity of *M. tuberculosis* pathogenesis and limited knowledge of the protective immune responses. The preclinical evaluation of novel tuberculosis vaccine candidates is also hampered by the lack of an appropriate animal model that could accurately predict the protective effect of vaccines in humans. Here, we review the role of zebrafish (*Danio rerio*) and other fish models in the development of novel vaccines against tuberculosis and discuss how these models complement the more traditional mammalian models of tuberculosis.

## Abstract (precis)

**Summary:** In this Review, we discuss how zebrafish (*Danio rerio*) and other fish models can complement the more traditional mammalian models in the development of novel vaccines against tuberculosis.

## Introduction

*Mycobacterium tuberculosis* (Mtb), the causative agent of tuberculosis (TB), has coexisted with the human population for thousands of years, causing high levels of mortality. Even today, despite extensive research and progress in the areas of prevention and treatment, TB remains the deadliest bacterial infection worldwide (Furin et al., 2019). According to the Global Tuberculosis Report 2019, 10 million new active disease cases are diagnosed each year, with 84% of the diagnoses confirmed in people living in 20 high-burden countries; for example, in regions of South-East Asia, Africa and the Western Pacific (WHO, 2019). Although TB affects all age groups in all parts of the world, the highest incidence is reported in low-income countries with a high human immunodeficiency virus (HIV) burden. Altogether, TB has been reported to be the cause of ∼1.5 million deaths each year and the most common cause of death in HIV-positive individuals (Furin et al., 2019; WHO, 2019). Active TB (see Glossary, Box 1) typically manifests as a pulmonary disease (Box 1) but more rarely, and especially among infants and immunocompromised people, a Mtb infection can progress into a disseminated disease, presenting, for example, as miliary or meningeal TB (Box 1) (Furin et al., 2019). In addition to active disease cases, 23% of the human population is estimated to carry Mtb in an asymptomatic and non-contagious form (Houben and Dodd, 2016). These ∼1.7 billion people with a latent Mtb infection (Box 1) have a lifetime risk of developing active disease and thus represent a considerable reservoir of potential disease. However, estimating the true burden of latent TB is challenging due to the limitations of the available diagnostic tests for TB, which currently comprise the tuberculin skin test (TST; Box 1) and the interferon γ (IFNγ) release assay (IGRA; Box 1) (Furin et al., 2019; Behr et al., 2018).

**Box 1. Glossary**

**Active tuberculosis:** the contagious form of the disease. Symptoms include fever, cough, bloody cough, weight loss and night sweats. Granulomas and tuberculous lesions in infected tissues are detected with x-ray.

**Adaptive immune response:** confers specific protection against pathogens days or weeks after exposure and is responsible for the antigen-specific immunological memory protecting against a secondary infection.

**Bacillus Calmette–Guérin** **(BCG):** an attenuated *M. bovis* used as a vaccine for tuberculosis.

**Caseating granuloma:** granuloma with a necrotic core (see ‘Necrotic granuloma core’) with caseating insides with a cheese-like appearance.

**Cellular immune response:** adaptive immune response mediated by CD4^+^ helper T cells that activate phagocytes and antigen-specific cytotoxic T cells (CD8^+^) to destroy intracellular pathogens or infected host cells.

**DNA vaccine:** a vaccine comprising a genetically engineered DNA construct encoding antigens that, after administration to the recipient, are expressed in host cells to elicit protective immune responses.

**Fibrotic capsule:** an epithelialized capsule built of fibroblasts that has formed around the infected cells to form a fibrous granuloma.

**Innate immune response:** non-specific and immediate defence mechanism comprising physiological barriers, receptors recognizing conserved patterns of foreign material, innate immune cells (such as macrophages, dendritic cells, granulocytes and natural killer cells), the complement system and inflammatory mechanisms.

**Interferon γ release assay (IGRA):** an assay to test the stimulation of leukocytes from a blood sample against tuberculosis antigens. Cannot distinguish between an active and latent infection.

**Latent tuberculosis:** non-contagious form of the disease. *M. tuberculosis* (Mtb) resides in granulomas without causing symptoms. Tuberculous granulomas can be visualized via x-ray. Latent disease can reactivate to an active infection in compromised immunity.

**Meningeal tuberculosis:** Mtb infection of the membranes enveloping the nervous system.

**Miliary tuberculosis:** Mtb infection that manifests across multiple tissues in ‘grain-like’ small lesions.

**Mtb** **virulence factor:** a molecule produced by Mtb that is essential for colonization and survival of the bacterium inside the host organism.

**Mucosa-associated lymphoid tissue (MALT):** lymphoid tissue of mucosa in various sites of the body, including the gastrointestinal tract, respiratory tract and urogenital tract. Rich in lymphocytes, macrophages and antigen-presenting mucosal cells (microfold cells, M-cells).

**Necrotic granuloma** **core:** the core of the granuloma contains dead and dying cells as a result of non-apoptotic cell death.

**Pulmonary tuberculosis**: Mtb infection in the lung.

**Recombinant BCG (rBCG):** a genetically modified *M.* *bovis* BCG strain.

**Th1:** a subtype of CD4^+^ T cells mediating a cellular immune response (see ‘Cellular immune response’).

**Th2:** a subtype of CD4^+^ T helper cells mediating protection; for example, against extracellular parasites and suppressing the Th1 cell response.

**Th17:** a subtype of CD4^+^ T helper cells mediating protection against intracellular pathogens through the recruitment of neutrophils and macrophages.

**Tuberculin skin test** **(TST):** test of reactivity to tuberculosis antigens. It is subject to false positives in BCG-vaccinated individuals, and cannot distinguish between an active and latent infection.

**Fig. 1.**
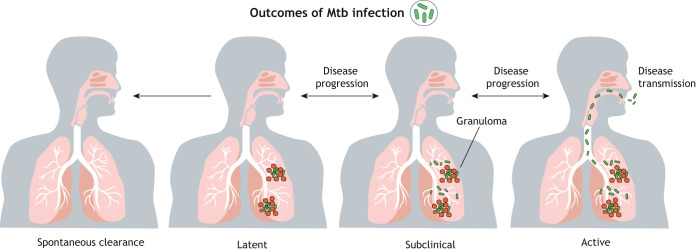
**Disease spectrum of pulmonary tuberculosis.** Pulmonary tuberculosis is the most common clinical manifestation of a *M. tuberculosis* (Mtb) infection. After primary infection, the disease can be spontaneously cleared by the immune system, remain latent, or progress into either subclinical or active infection. In latent, subclinical or active infection, Mtb (green) persist in the lung tissues in granulomas (red). In subclinical and active infection, free Mtb also reside in the lungs. In active disease, free Mtb are also secreted into the airways, which makes active disease contagious. Latent infection can progress into subclinical or active disease, and subclinical disease can progress into active disease, meaning that carriers of latent TB represent a significant disease reservoir.

Although the clinical presentation of TB has traditionally been divided into acute and latent infections, the overall disease spectrum of TB is highly variable (Fig. 1) (Drain et al., 2018; Cadena et al., 2017). In humans, the clinical symptoms of active TB include persistent cough, blood-stained sputum, weight loss and fever. Disease transmission occurs during the active phase through aerosol droplets containing Mtb (Sia and Rengarajan, 2019). TB is a granulomatous inflammatory disease, characterized by the presence of Mtb-containing immune cell clusters – granulomas – at the site of infection, mostly the lungs, but also in the lymph nodes and elsewhere in the body when the disease becomes disseminated (Sia and Rengarajan, 2019; Cadena et al., 2017). Granulomas form around macrophages infected with Mtb to prevent the dissemination of bacteria, but, at the same time, they are also the predominant site for Mtb replication and dissemination (Cadena et al., 2017). During the latent infection, bacteria inside the granulomas may become metabolically inactive, allowing them to persist in the human body for decades (Peddireddy et al., 2017). Even during this seemingly static and asymptomatic phase, the host-pathogen interactions in granulomas are highly dynamic, and the fate of infection (Fig. 1) is thought to be a result of continuous interplay between Mtb and its host (Cadena et al., 2017). Approximately 5-10% of the individuals with a latent infection develop active TB (WHO, 2019; Behr et al., 2018).

Owing to the complex pathophysiology and an incomplete understanding of the definitive steps leading to progression of the infection or reactivation of the latent disease, the outcome of an Mtb infection is poorly predictable. The conventional treatment of TB is a combination of four antibiotics – isoniazid, rifampin, pyrazinamide, and either ethambutol or streptomycin (Furin et al., 2019) – which requires long-term drug regimens lasting 6-9 months and is associated with poor patient compliance and frequent treatment failure (Furin et al., 2019; Munro et al., 2007). This also creates an excellent environment for the development of antibiotic-resistant Mtb, which has become a major problem in areas with a high TB incidence such as India, China and Russia (WHO, 2019). Although the global incidence and mortality are declining, the burden of TB in low-income countries remains high (WHO, 2019). In these countries, early and comprehensive diagnosis and treatment are hampered by the poor availability of health services, and, as a consequence, from the estimated 10 million annual cases of TB, only 6.4 million are officially diagnosed and properly treated (WHO, 2019).

A more economical and practical approach for controlling TB is to prevent infections through vaccination. However, owing to variation in the protective efficiency of the current TB vaccine, Bacillus Calmette–Guérin (BCG; Box 1), the goal of eradicating TB has not been achieved. While BCG protects young children from meningeal and miliary TB, it fails to prevent primary Mtb infections or latent disease reactivation (Dockrell and Smith, 2017; Mangtani et al., 2014). In addition, estimates of the effect of BCG on pulmonary TB vary significantly, ranging from 0% to 80% (Mangtani et al., 2014). Even though BCG protection reportedly lasts for up to 50 years (Aronson et al., 2004), in most cases, the protective effect wanes by adolescence, leaving the adult population poorly protected (Mangtani et al., 2017).

To address the shortcomings of the BCG vaccine and the problem of drug-resistant Mtb, a concerted research effort is underway to develop new anti-Mtb vaccines, and several vaccine candidates are currently in phases I-III of clinical trials (Table 1; also reviewed in detail in, for example, Hatherill et al., 2020; Martin et al., 2020). Although recent success with BCG revaccination, conferring 45.5% protection in previously BCG-vaccinated healthy adolescents (Nemes et al., 2018), paves the way for the success of other TB vaccines, many of the candidates that show protection in animal models fail in clinical trials. Therefore, reliable preclinical models with predictive value for human trials are needed to improve efficacy and save costs and time in vaccine development.

**Table 1.**
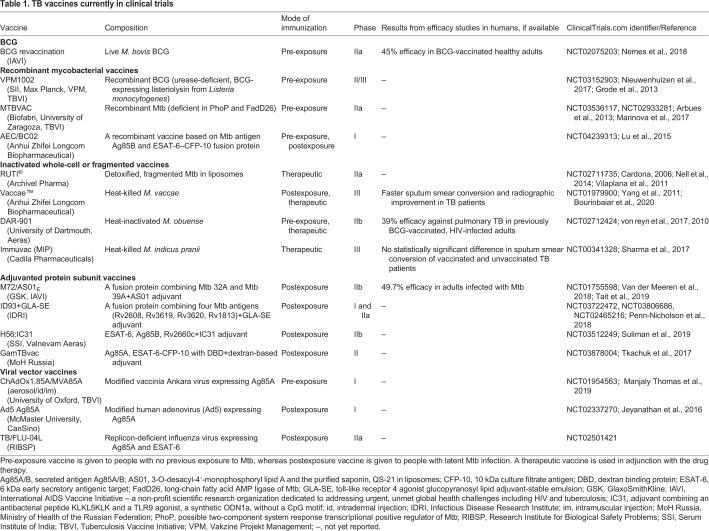
**TB vaccines currently in clinical trials**

Mice are by far the most used model in the evaluation of novel TB vaccines, followed by other common mammalian models: guinea pig, rabbit, cattle and non-human primates (NHPs) (reviewed in Gong et al., 2020; Myllymäki et al., 2015). However, small mammalian models are limited by their lack of natural susceptibility to mycobacterial infection, whereas NHPs raise ethical concerns (Gong et al., 2020; Myllymäki et al., 2015). As an alternative to mammalian models, fish models, and especially zebrafish (*Danio rerio*), have gained popularity in TB vaccine development. Fish are naturally susceptible to *Mycobacterium marinum*, a close relative to Mtb that causes a disease resembling human TB (Hashish et al., 2018; Tobin and Ramakrishnan, 2008). The related pathogenesis of and host responses to Mtb and *M. marinum* infections, combined with the suitability of fish for large-scale antigen screens, hold promise for this model in supporting the preclinical evaluation of novel TB vaccine candidates.

In this Review, we discuss how fish models, especially the zebrafish, can complement the more traditionally used mammalian models for the development of novel TB vaccines. We start with a brief introduction to the common mammalian models of TB and discuss their advantages and limitations in light of recent data from preclinical and clinical studies. Finally, we highlight the potential of the zebrafish and other fish models in TB vaccine development by discussing the latest achievements made with these models.

## Mammalian models of TB

Prior to clinical trials, the safety, immunogenicity and protective effect of each vaccine candidate is evaluated in animal models. The safety of the vaccine candidate is a prerequisite for progressing to clinical trials, while immunogenicity studies provide insights into proper vaccination schedules and doses for efficacy studies. The efficacy of candidate TB vaccines is tested in Mtb challenge studies, where protection in vaccinated animals is measured by, for example, survival, Mtb burden or the pathology score (McShane and Williams, 2014). In general, a vaccine candidate must demonstrate improved protection compared to BCG to progress to clinical trials. Owing to the complex pathophysiology of human TB, it is challenging to model all its aspects in one animal species and, therefore, TB vaccine candidates are preclinically validated in several species. In the following section and in Table 2, we briefly introduce the main mammalian models used in TB research.

**Table 2.**
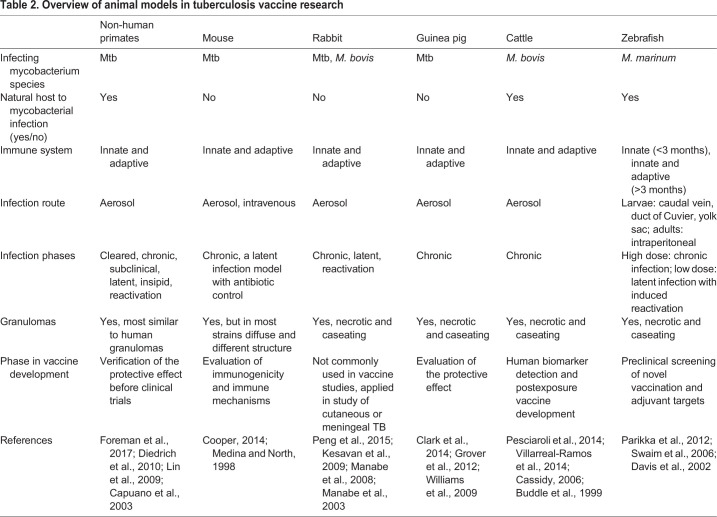
**Overview of animal models in tuberculosis vaccine research**

### Mouse

The mouse is the most widely used model in TB vaccine development. Mice are relatively cost efficient, easy to maintain, and are supported by well-established genetic and immunological tools (Cooper, 2014). An Mtb infection in mice can be induced by aerosol, resulting in an active infection with a stable high Mtb burden and early death without a latent phase (Cooper, 2014). As Mtb is not a natural murine pathogen, many of the commonly used mouse strains (such as BALB/c and C57Bl/6) are resistant (Medina and North, 1998). Granulomas in mice also differ from those in humans as they lack necrotic cores (Box 1) and organized structure (Orme and Basaraba, 2014; Rhoades et al., 1998). To overcome these limitations and to better mimic the pathophysiology of human TB, researchers use immunodeficient or genetically modified strains, such as C3HeB/FeJ mice, which exhibit caseating granulomas (Box 1) (Lanoix et al., 2015; Driver et al., 2012). However, these lines show varying susceptibilities and lung pathologies, and thus different responses to drugs and vaccines, complicating the extrapolation of the results to humans. Despite their limitations, mice are frequently used in preclinical studies, where they offer a practical tool for evaluating the immunogenicity and the mechanisms of protection of novel TB vaccine candidates (Cardona and Williams, 2017). However, verification of the protective effect of the candidate in other models, which better recapitulate the complexity of human TB, is required.

### Guinea pigs

Guinea pigs are well suited for TB research. Although they are not natural hosts, guinea pigs are highly susceptible to Mtb via the airways and recapitulate the main features and symptoms of the acute TB in humans (Clark et al., 2014). Granulomas in Mtb-infected guinea pigs are similar to human granulomas, with corresponding cell types, necrotic cores and fibrotic capsules (Box 1) (Turner et al., 2003). However, an Mtb infection in guinea pigs is always progressive and fatal, and exhibits no latent phase (Clark et al., 2014; Grover et al., 2012; Williams et al., 2009). Compared to mice, higher costs and maintenance requirements, as well as scarcity of immunological reagents and genetic tools, limit the use of guinea pigs in TB research. In TB vaccine research, guinea pigs are the preferred small mammals for testing the protective effects of novel TB vaccine candidates. Owing to the lack of latency, however, this model is not suited for studying vaccines against the reactivation of latent TB.

### Rabbit

Mtb and *Mycobacterium bovis* infections have been modelled in rabbits and, when introduced via the airways, both result in a spectrum of diseases similar to human TB (Peng et al., 2015). Although the ability to resist infection depends on the Mtb strain used (Manabe et al., 2003), rabbits are not particularly susceptible to Mtb and can contain the infection in a latent form that can then be experimentally activated by a corticosteroid treatment to model TB reactivation (Kesavan et al., 2009; Manabe et al., 2008). Similar to guinea pigs, the histology of granulomas in rabbits, with their necrotic, caseous cores, resembles that in humans. However, the use of rabbits in TB research is hampered by the lack of reagents and tools for research, as well as by high costs, poor availability of animals and ethical concerns (Peng et al., 2015). Although the presence of the entire disease spectrum of an Mtb infection in rabbits is a considerable advantage over other small mammalian models, rabbits are only selectively used in vaccine development and are more often utilized to study the rarer forms of human TB, cutaneous and meningeal TB (Peng et al., 2015).

### NHPs

As the genetically closest relatives to humans and natural hosts for Mtb, NHPs are the best models for human TB (Foreman et al., 2017). All aspects of human TB can be modelled in NHPs due to the similarities in immune response, disease pathology and clinical manifestation (Foreman et al., 2017; Capuano et al., 2003). Most importantly, NHPs recapitulate the whole spectrum of human TB and show similar heterogeneity in disease outcomes and granuloma pathology at the individual level (Lin et al., 2009; Capuano et al., 2003). One of the biggest advantages of NHPs is their ability to develop a long-lasting latent infection, although only about 40% of the infected individuals develop latency while the rest acquire an active disease (Lin et al., 2009; Capuano et al., 2003). Furthermore, reactivation of latent TB can be studied in a setting of simian immunodeficiency virus co-infection, thus mimicking the HIV-positive human population with a high TB risk (Diedrich et al., 2010). Although the advantages of NHPs as TB models are numerous, their use is limited due to notable economical and ethical constraints. In the preclinical evaluation of TB vaccine candidates, NHP models are invaluable for the verification of promising results obtained from smaller animals prior to progressing to clinical trials, but their use in early-phase examination is unjustified.

### Cattle

*M. bovis* is a close relative of Mtb and a natural pathogen of cattle and other domestic animals (Pesciaroli et al., 2014). Only a few bacilli can cause infection in susceptible hosts and the resulting disease is highly similar to human TB (Cassidy, 2006). As in humans, BCG confers partial protection against *M. bovis* in cattle and is used to prevent the disease and the associated economic losses in farmed animals and wildlife reservoirs (Villarreal-Ramos et al., 2014). For this reason, together with the similar immune responses observed in cattle and humans, cattle can be used in the development of TB vaccines. As other models, however, cattle have their drawbacks, including high costs and maintenance requirements, as well as the ethical and availability concerns of using these animals for research purposes.

### Mammalian models in preclinical evaluation of TB vaccines

Preclinical evaluation of several promising candidates is currently underway. Many of the novel strategies are based on recombinant BCGs (rBCG; Box 1) to improve the somewhat poor protection elicited by parenteral BCG. For example, an rBCG with a deletion in the Rv0198 gene locus encoding Zmp1 (see Box 2 for the full names of mycobacterial antigens discussed in this Review) has been evaluated in several animal models with encouraging results; compared to parenteral BCG, rBCGΔzmp1 conferred enhanced immunogenicity in mice and cattle and improved protection in guinea pigs (Sander et al., 2015; Khatri et al., 2014; Johansen et al., 2011). Another rBCG expressing the ESX-1 secretion system from *M. marinum* (BCG::ESX-1^Mmar^) showed improved protection compared to BCG in mice, as subcutaneous immunization decreased Mtb loads in the spleen and lungs after aerosol Mtb infection (Gröschel et al., 2017). Similarly, a recombinant BCG expressing the *Escherichia coli* heat-labile enterotoxin was more effective at reducing the bacterial burden in the murine lung compared to BCG (Nascimento et al., 2017).

**Box 2. Mycobacterial genes and proteins mentioned in the text**

**Ag85:** secreted antigen Ag85

**Cdh:** CDP-diacylglycerol pyrophosphatase

**CFP-10:** 10 kDa culture filtrate antigen CFP-10

**ESAT-6:** 6 kDa early secretory antigenic target

**ESX-1:** 6 kDa early secretory antigenic target secretion system

**MMAR_4110:** an aldehyde dehydrogenase of *M. marinum*

**MMAR_4207:** conserved hypothetical membrane protein of *M. marinum*

**PE_31:** a PE protein family member possessing the characteristic N-terminal PE domain

**PE5_1:** a PE protein family member possessing the characteristic N-terminal PE domain

**PPE15:** a PPE protein family member possessing the characteristic N-terminal PPE domain

**RD4:** region of difference 4 locus

**RpfB:** resuscitation-promoting factor B

**RpfE:** resuscitation-promoting factor E

**Rv1016c:** Mtb gene locus encoding a probable conserved lipoprotein LpqT

**Zmp1:** probable zinc metalloproteinase

Animal studies have also provided important insights into the relevant delivery routes of TB vaccines. The standard routes of immunization in mammals and humans include intradermal and intramuscular, which aim to elicit systemic immune responses (Stylianou et al., 2019; Dijkman et al., 2019; Brandt et al., 2004; Skeiky et al., 2004). However, in 1973, a study in NHPs showed that mucosal vaccination could enhance protection against airborne Mtb (Barclay et al., 1973). Since then, many other studies have supported the important role of mucosally activated lymphoid tissues (MALTs; Box 1) in protecting against a mycobacterial infection; for example, in mice (Aguilo et al., 2014) and in guinea pigs (Garcia-Contreras et al., 2008), although some also showed contradictory results, as in rhesus macaques (Sibley et al., 2016). The most recent evidence of the importance of a local immune response in clearing Mtb comes from an early-phase efficacy study in mice, which revealed that boosting BCG with ChadOx1.PPE15, a replication-deficient chimpanzee adenovirus expressing the Mtb antigen PPE15, elicited better protection against an aerosol Mtb infection in mice compared to BCG without a booster. However, the enhanced protection was only evident in mice immunized intranasally, but not in the intradermally vaccinated ones (Stylianou et al., 2018). Correspondingly, rhesus macaques immunized with BCG by endobronchial installation had reduced lung pathology and bacterial counts in the lungs compared to animals immunized with intradermal BCG (Dijkman et al., 2019). Interestingly, BCG vaccination of rhesus macaques through the intravenous route has been shown to elicit superior protection to intradermal or aerosol immunization (Darrah et al., 2020; Barclay et al., 1970). In a recent study by Darrah et al. (2020), intravenous immunization by BCG resulted in as much as a 100,000-fold reduction in thoracic Mtb count compared to intradermal administration, with six out of 10 animals having no detectable Mtb in any tissue analysed compared to 10/10 with signs of infection in the intradermal and aerosol immunization groups. Although perhaps not applicable as such in humans, these results – and the model used – provide important clues on the mechanisms of protection against TB.

The above-mentioned mammalian models are indispensable for the development of a novel TB vaccine, although, as noted, only NHPs can fully recapitulate the aspects of human TB. As we still have relatively limited data from clinical trials, the predictive value of each animal model has yet to be fully determined. Some TB vaccine candidates have shown good correlation between animal and human studies, as is the case for the candidate M72/AS01_E_ (Tait et al., 2019; Van der Meeren et al., 2018), which was recently reported to confer 47.9% protection in otherwise healthy Mtb-infected adults in a phase IIb trial (Tait et al., 2019) (Table 1). This success was preceded by preclinical safety and efficacy evaluation in mice, guinea pigs, rabbits and NHPs (Reed et al., 2009; Tsenova et al., 2006; Brandt et al., 2004; Skeiky et al., 2004). Particularly, the BCG prime-M72/AS01E boost strategy significantly reduced bacterial loads in the rabbit model of TB meningitis (Tsenova et al., 2006) and improved protection compared to BCG in cynomolgus macaques (Reed et al., 2009). However, in many cases, vaccines with an acceptable safety and efficacy profile in animals fail to show any effect in humans, as exemplified by the candidate MVA85A, a modified vaccinia Ankara virus expressing the Mtb antigen Ag85A (Tameris et al., 2013). At the preclinical stage, researchers observed improved protection elicited by MVA85A compared to BCG in mice and, as a booster for BCG, in guinea pigs and cattle (Vordermeier et al., 2009; Verreck et al., 2009; Williams et al., 2005; Goonetilleke et al., 2003). Conversely, although the BCG prime/MVA85A booster strategy in NHPs decreased the bacterial burden in the lungs, it did not improve protection compared to BCG (Verreck et al., 2009). The results from the MVA85A trial, although disappointing, evoked a conversation about the importance of not only the animal chosen, but also of optimizing the experimental setting – including Mtb strain, infection route, environmental factors – to better correspond to the situation in clinical trials (reviewed in detail in McShane and Williams, 2014).

As discussed in previous sections, no model is perfect, but they all serve specific purposes on the way to vaccine development from design to clinical translation (Table 2). As an alternative to mammalian models, several fish models, and particularly the zebrafish (*Danio rerio*), have recently been used in TB vaccine research. These vertebrate species provide practical, ethical and reliable models for conducting large-scale screens for TB vaccine antigens, as we discuss below.

## Zebrafish as a model for TB

The zebrafish model for TB was introduced in the early 21st century (Davis et al., 2002) and utilizes the natural relationship of the fish with *M.* *marinum*, a close relative of Mtb (Hashish et al., 2018; Tobin and Ramakrishnan, 2008). A *M. marinum* infection in zebrafish shares many similarities in its pathogenesis and host responses with human Mtb infection, including the whole spectrum of TB disease, as we discuss in the next section.

Zebrafish offer several advantages for modelling TB infections. They are easy to maintain and to modify genetically and can be used in large batches for experiments (Luukinen et al., 2018; Benard et al., 2012). Importantly, when using the fish pathogen *M. marinum* instead of Mtb, there is no need for biosafety considerations, which decreases the costs and space demands for the studies. Zebrafish embryos are optically transparent, and the availability of transgenic zebrafish lines with fluorescently labelled components of the immune system enable the observation of the responses of zebrafish embryos to a *M. marinum* infection in real time (Meijer and Spaink, 2011). However, zebrafish embryos fight a Mtb infection only with their innate immune system, and while this model provides an opportunity to study the function of the innate immune response (Box 1) without the influence of adaptive responses, it is not useful in vaccination studies. Adult zebrafish, on the other hand, possess a fully developed adaptive immune response (Box 1) and an inducible protective immunological memory against *M. marinum*, rendering them suitable for vaccine development (Swaim et al., 2006; Parikka et al., 2012; Oksanen et al., 2013).

The lack of lungs and lymph nodes in zebrafish is a considerable limitation and prevents the study of the disease pathology at these primary sites of human TB. Differences between the immune systems of zebrafish and humans, including different classes of antibodies in fish (IgM, IgD and the fish-specific IgZ) and humans (IgG, IgM, IgD, IgE and IgA), the expansion of innate immune system-related genes due to the genome duplication in teleost fish, and the lack of basophilic neutrophils in zebrafish, might also affect disease pathology and host responses and, therefore, the immunogenicity and protective effect of a candidate vaccine (Stein et al., 2007; Danilova et al., 2005; Bennett et al., 2001). Additionally, although the natural relationship between fish and *M. marinum* is attractive for TB research, it must be noted that *M. marinum* and Mtb are not genetically identical and not all of the antigens are shared between the two species (Stinear et al., 2008; Tobin and Ramakrishnan et al., 2008). Finally, the limited availability of immunological reagents, such as zebrafish-specific antibodies, hampers the immunogenicity evaluation of novel vaccine candidates.

Next, we discuss the zebrafish as a model for developing a TB vaccine and summarize the latest advances achieved using this model. We begin by briefly discussing the related pathogenesis and host responses in *M. marinum* and Mtb infections.

### *M. marinum* infection in zebrafish

*M. marinum* represents the genetically closest relative of Mtb (Stinear et al., 2008; Tobin and Ramakrishnan, 2008) and is a common cause of TB disease in many ectotherms, such as frogs and fish (Hashish et al., 2018). The genes associated with *M. marinum* and Mtb pathogenesis are highly conserved and both pathogens exploit similar changes in genetic programmes to promote different disease stages in their hosts, again indicating related pathogenic mechanisms (Stinear et al., 2008; Tobin and Ramakrishnan, 2008). As a result, the chronic, progressive granulomatous infection caused by *M. marinum* is highly similar in its histopathology to human TB (Hashish et al., 2018)*.* In adult zebrafish, depending on the bacterial dose, an experimental *M. marinum* infection can progress to an acute (high dose) or chronic (low dose) disease (Luukinen et al., 2018; Swaim et al., 2006; van der Sar et al., 2004; Prouty et al., 2003). *M. marinum* granulomas in zebrafish are well organized and histologically highly similar to Mtb granulomas, with their necrotic centres surrounded by infected macrophages, epithelial cells and infiltrating CD4^+^ cells and neutrophils (Yoon et al., 2015; Yang et al., 2012; Swaim et al., 2006; van der Sar et al., 2004). Zebrafish granulomas also recapitulate other important aspects of human granulomas, including hypoxia and the influx and efflux of naïve and infected macrophages (Myllymäki et al., 2018a; Oehlers et al., 2015; Davis et al., 2002). Importantly, a low-dose *M. marinum* infection in zebrafish proceeds into the latent phase, characterized by necrotic and hypoxic granulomas with centrally organized bacteria and the ceasing of bacterial growth (Myllymäki et al., 2018a; Parikka et al., 2012). The reactivation of a latent *M. marinum* infection in zebrafish can be induced by γ-irradiation or by treatment with an immunosuppressive agent, leading to the disruption of granulomas and the loss of control of bacterial growth (Myllymäki et al., 2018a; Parikka et al., 2012).

The early events of the host-pathogen interaction, including bacterial recognition and phagocytosis by macrophages, bacterial evasion of phagolysosomal killing and granuloma formation are shared between Mtb and *M. marinum* (reviewed, for example, in Hodgkinson et al., 2019; Myllymäki et al., 2016), and have been extensively studied in zebrafish embryos. The adaptive immune response, however, is less explored in zebrafish. In humans, the adaptive immune response to Mtb is thought to be mainly mediated by the cellular immune response (Box 1). For their role in activating the antimicrobial mechanisms of macrophages, IFNγ- and tumour necrosis factor (TNF)-secreting CD4^+^ cells of the Th1 (Box 1) subset have been shown to be the predominant cell type controlling Mtb in humans and animal models (Cooper et al., 1997, 1993; Flynn et al., 1993). Challenge studies in mutant zebrafish that lack active lymphocytes [*recombination-activating gene 1* (*rag1*)*^−/−^*] have confirmed that the control of an active and latent *M. marinum* infection depends on functional lymphocytes (Myllymäki et al., 2018a; Hammarén et al., 2014; Parikka et al., 2012; Swaim et al., 2006), and the presence of CD4^+^ cells in granulomas suggests an important role for these cells in constraining a *M. marinum* infection (Yoon et al., 2015). In addition, an enhanced Th1 response was associated with reduced bacterial growth and improved survival in Interleukin 10 (Il10) mutant zebrafish with a progressive *M. marinum* infection (Harjula et al., 2018).

So far, most of the vaccine development has concentrated on evoking strong Th1 responses, although human and animal studies have provided conflicting data on the matter. When its efficacy as a booster for BCG was tested in phase IIb clinical trials, the vaccine candidate MVA85A induced long-lived CD4^+^ cells expressing IFNγ, TNF and IL2, but this response did not correlate with protection (Tameris et al., 2013). Correspondingly, no association between the elicited Th1 response and protection was seen in infants vaccinated with BCG (Kagina et al., 2010). In light of these and other studies, it is therefore evident that although a Th1/IFNγ response is essential for protection against mycobacteria, other mechanisms, mediated by CD8^+^, unconventional and regulatory T cells, and by B cells, also play important roles (as reviewed in detail in Cardona and Cardona, 2019; Lu et al., 2016; Behar, 2013). It also means that correlates of protection, other than the currently used IFNγ, would be valuable in the preclinical and clinical evaluation of TB vaccine candidates. A recent study in NHPs highlighted the role of the Th17 (Box 1) response, as the protection mediated by pulmonary immunization of rhesus macaques with BCG was associated with increased levels of Th17 cells but not with IFNγ production (Dijkman et al., 2019). In addition, owing to the growing evidence from animal and human studies, there is now a consensus that the importance of the synergistic effect of Th1 and Th2 (Box 1), instead of Th1 alone, in fighting Mtb might be underappreciated and should not be neglected in the development of TB vaccines (Abebe, 2019; Loxton, 2019). Supporting this, an improved Th2 response was associated with better bacterial control during latency in the zebrafish-*M. marinum* model (Hammarén et al., 2014).

As noted above, a complete understanding of the mechanisms of protection in human Mtb infection is currently lacking, yet crucial for the development of novel vaccines. The zebrafish-*M. marinum* model, in which both the active and latent phase of the mycobacterial infection can be studied, provides a feasible platform. Along with the above-mentioned studies on the adaptive immune response to *M. marinum* infection, several transcriptome analyses of wild-type (Harjula et al., 2020; Ojanen et al., 2019; Hegedűs et al., 2009; van der Sar et al., 2009) and a hypersusceptible mutant (Harjula et al., 2020) zebrafish have also provided invaluable insights into host responses and the factors that affect host susceptibility to mycobacterial infection. In addition to providing insights into the protective mechanisms in human TB, adult zebrafish can also be used for the direct assessment of novel TB vaccine candidates and delivery methods, as we discuss next.

## Modelling TB vaccine development in zebrafish

**Fig. 2.**
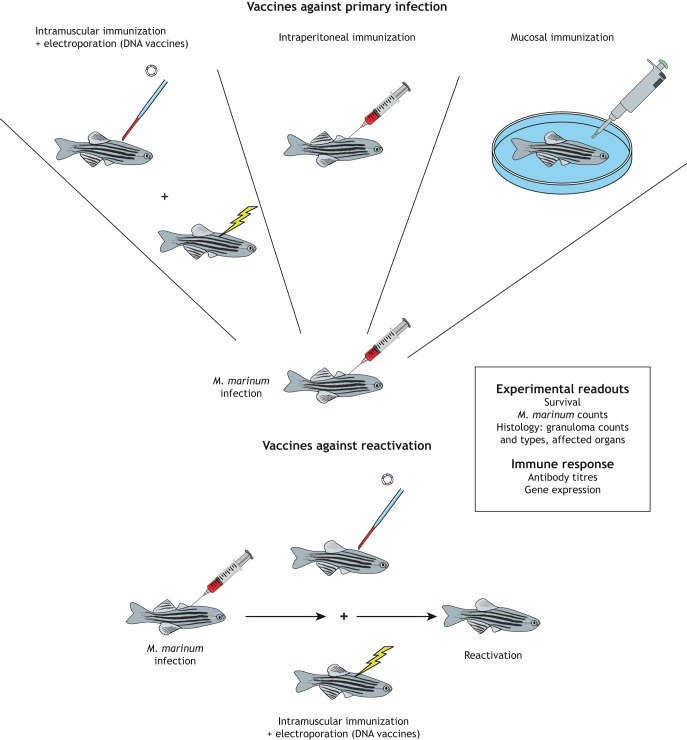
**Vaccination routes and methods in zebrafish.** Zebrafish are usually infected by *M. marinum* via intraperitoneal injection or immersion. Three immunization routes (intramuscular, intraperitoneal and mucosal) are used in zebrafish to evaluate a vaccine's effect on the primary infection (upper panel). With DNA vaccines, the intramuscular injection of the expression construct is followed by electroporation of the target tissue. To assess a vaccine's effect on the reactivation of a latent *M. marinum* infection, zebrafish with a latent infection are vaccinated (using an intramuscular injection of a DNA vaccine followed by electroporation), after which the latent infection is activated by treatment with the immunosuppressant dexamethasone (lower panel). The figure also depicts the methods commonly used to evaluate the protective effect and the immunogenicity of the candidate vaccines in zebrafish.

As described above, a *M. marinum* infection in zebrafish recapitulates the disease spectrum of human TB, allowing the study of novel vaccines against both the primary infection and the reactivation of a latent infection. In particular, large-scale screens to find the most immunogenic and protective Mtb antigen or antigen combination can be conducted in the ethical and cost-efficient zebrafish model. Zebrafish are usually vaccinated via intraperitoneal or intramuscular injections, although mucosal immunization by immersion can also be performed (Fig. 2) (Risalde et al., 2018; Oksanen et al., 2013). In the absence of biomarkers and reagents to assess the immunogenicity of candidate antigens in zebrafish, improved infection control reflected by survival rate, bacterial counts, number of granulomas and affected organs are used to evaluate the potential of vaccine candidates (Myllymäki et al., 2018a; Oksanen et al., 2013). Compared to inbred mice strains, zebrafish are genetically heterogeneous in laboratory conditions and the ability of individuals to resist *M. marinum* usually varies, but this can be overcome by using adequate group sizes based on power calculations (Balik-Meisner et al., 2018). Also, this recapitulates the natural diversity in the human population (Balik-Meisner et al., 2018; Churchill et al., 2004). The studies evaluating the efficacy of BCG and novel vaccine candidates in zebrafish are summarized in the following section and in Table 3.

**Table 3.**
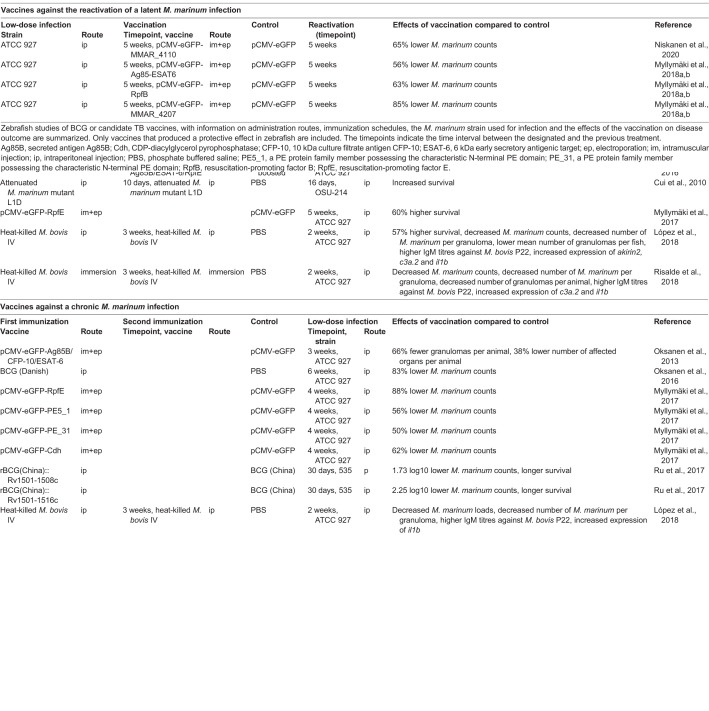
**Vaccines against acute, chronic** **or reactivated latent** ***M. marinum*** **infections in zebrafish**

### The protective effect of BCG in zebrafish

As the standard control for the preclinical and clinical evaluation of vaccine candidate efficacy, the effects of BCG against acute (high dose) or chronic (low dose) *M. marinum* infection have been tested in zebrafish. As with human Mtb, BCG does not prevent *M. marinum* infection but confers modest and variable protection in studied individuals (Su et al., 2019; Ru et al., 2017; Oksanen et al., 2016, 2013). This modest protection in zebrafish is likely to be associated with the poor replication of the BCG strain of *M. bovis* in zebrafish (Oksanen et al., 2016). Immunization with BCG improves the survival of zebrafish with acute *M. marinum* infection and lowers bacterial counts in chronic *M. marinum* infection (Oksanen et al., 2016, 2013). Importantly, this protective effect is enhanced by a booster DNA vaccination, which encodes the antigen combination Ag85B, ESAT-6 and RpfE (Oksanen et al., 2016). While we lack a complete understanding of the immunological mechanisms behind this protective effect in zebrafish, BCG is known to induce Th1-biased IL17-dependent responses in humans and in animal models (Gopal et al., 2012; Pitt et al., 2012; Umemura et al., 2007). Correspondingly, BCG injection in zebrafish induced the expression of several genes of the innate and adaptive immune responses, including those encoding Il17 and its receptor (Oksanen et al., 2016). The improved control of a *M. marinum* infection in vaccinated fish has also been associated with a restricted inflammatory response, mediated by TNF (Oksanen et al., 2016).

### Evaluating novel TB vaccine candidates in zebrafish

#### Recombinant BCG and other whole-cell vaccines

Vaccines based on live, recombinant or attenuated mycobacteria represent one of the most successful strategies for TB vaccine development, as demonstrated by their prevalence among the novel TB vaccine candidates at clinical trials (Table 1). These include the recombinant BCG VPM1002 (phase III) (Nieuwenhuizen et al., 2017) and recombinant Mtb MTBVAC (phase IIa) (Arbues et al., 2013), which improved the protective immune response compared to BCG in preclinical studies and are currently being evaluated in humans (Table 1). Complementing this success, zebrafish immunized with a BCG strain expressing genes of the virulence-associated region of difference (RD) 4 locus from Mtb had higher survival rates and lower bacterial counts in a chronic *M. marinum* infection compared to infected fish immunized with parental BCG (Ru et al., 2017). Conversely, immunization of zebrafish with BCG expressing a mannosylated protein of Mtb encoded by the locus Rv1016c conferred decreased protection against *M. marinum* compared to immunization with parental BCG (Su et al., 2019). This poor protection compared to BCG was associated with impaired Th1 and Th17 responses and dendritic cell maturation in mice, suggesting that Rv1016c is an important Mtb virulence factor (Box 1) that allows escape from the host's immune system (Su et al., 2019).

Along with recombinant BCG, other whole-cell vaccines, such as heat-killed *Mycobacterium* *vaccae* (Vaccae^TM^; Bourinbaiar et al., 2020), *Mycobacterium* *obuense* (DAR-901; von reyn et al., 2017) or *Mycobacterium* *indicus pranii* (Immuvac; Sharma et al., 2017), or fragmented Mtb (RUTI; Nell et al., 2014), have advanced to clinical trials (Table 1). In zebrafish, heat-killed *M. bovis* and attenuated *M. marinum* elicit a protective immune response against chronic and acute *M. marinum* infection (López et al., 2018; Risalde et al., 2018; Cui et al., 2010). Interestingly, heat-killed *M. bovis* protected zebrafish against a *M. marinum* infection through mucosal vaccination by immersion, indicating that this clinically important vaccination route for a TB vaccine is also applicable to zebrafish (Risalde et al., 2018). In both systemic and mucosal immunization of zebrafish by heat-inactivated *M. bovis*, the protection was associated with the production of anti-*M. bovis* antibodies and with the increased expression of two genes related to the innate immune response, *interleukin 1b* (*il1b*) and *complement component 3a, duplicate 2* (*c3a.2*).

#### DNA vaccines

In addition to its poor efficiency, the use of BCG is also hampered by the risk of a disseminated infection in immunocompromised individuals, especially HIV-infected children (Hesseling et al., 2007; WHO, 2019), driving research into safer alternatives. Subunit vaccines, consisting, for example, of purified proteins or virus-like particles, or DNA vaccines (Box 1), are both safer than live vaccines and enable the introduction of multiple antigens to broaden the coverage and to direct the immune response to desired pathways (Hobernik and Bros, 2018; Suschak et al., 2017). Importantly, DNA vaccines also have less stringent storage requirements relative to other vaccine types, making them amenable for use in all parts of the world (Suschak et al., 2017). In the case of TB, DNA vaccines are also particularly useful in providing Mtb antigens that are expressed during different metabolic states and thus confer protection against different disease stages (Hobernik and Bros, 2018; Khademi et al., 2018). Owing to their potential, several DNA vaccines based on the most common Mtb virulence factors, including ESAT-6, Ag85 and PE/PPE family members (Box 2), combined with novel acute-phase or latency-associated antigens, have recently been tested in murine models (e.g. Liang et al., 2018, 2017; Tang et al., 2018). Although they have shown promise in their immunogenicity and protective effect in mice, no DNA vaccine against TB is currently in clinical trials. In fact, although DNA vaccines have been approved for veterinary use; for example, a horse vaccine against West Nile Virus and a salmon vaccine against the infectious hematopoietic necrosis virus (e.g. Collins et al., 2019), no DNA vaccine has yet been licensed for clinical use in humans (Collins et al., 2019; Hobernik and Bros, 2018).

Various combined DNA vaccines have been shown to protect zebrafish from acute and chronic *M. marinum* infections, including a DNA vaccine combining the Mtb antigens Ag85B, CFP-10 and ESAT-6 (Oksanen et al., 2013) and one that combined Ag85B, ESAT-6 and RpfE, which boosted the protective effect of BCG (Oksanen et al., 2016). To demonstrate the suitability of the zebrafish model for preclinical screening of novel TB vaccine antigens, Myllymäki et al. (2017) screened 15* M. marinum* antigens in zebrafish for their protective efficacy against a primary *M. marinum* infection. These antigens have Mtb counterparts with immunogenic properties in mice and/or humans, and were used in a single dose to prime the immune response against a chronic or acute *M. marinum* infection. The screen consisted of intramuscular injection of a plasmid encoding an antigen–GFP fusion protein with adequate antigen expression in the dorsal muscle. Expression of the PE5_1, PE_31, RpfE or Cdh antigens improved infection control in a chronic *M. marinum* infection. One antigen, RpfE, also improved the survival of zebrafish with acute infection.

Owing to the high burden of latent TB, a vaccine that offers protection against reactivation would have a major impact. However, in the absence of a proper model for latent and reactivating Mtb infection, the studies that focus on these disease stages, and on the development of a vaccine that targets reactivation, have been limited. The zebrafish offers hope in this regard, with its naturally developing disease latency and spontaneous reactivation of a mycobacterial infection. To identify protective antigens against reactivation, 15* M. marinum* antigens were screened in zebrafish for their protective effect against reactivation of a latent *M. marinum* infection (Myllymäki et al., 2018a). Fish with a latent infection were injected with a DNA vaccine expressing selected antigens prior to *M. marinum* reactivation by dexamethasone treatment. This screen revealed that two antigen candidates, RpfB and MMAR_4207, and the antigen combination of Ag85 and ESAT-6, confer partial protection against reactivation (Myllymäki et al., 2018a). Thus, this study identified two novel antigen candidates and provided support for the use of zebrafish to investigate TB vaccines that prevent reactivation of a latent infection. In a recent study by Niskanen et al. (2020), an *in vitro* model for *M. marinum* latency and reactivation was used to identify mycobacterial genes specifically expressed during reactivation. When seven of these were tested as vaccine antigens, MMAR_4110 was shown to be protective against the reactivation of *M. marinum* infection in zebrafish (Niskanen et al., 2020). Although MMAR_4110, an aldehyde dehydrogenase, does not have a clear homologue in Mtb, the results suggest that the alcohol hydrogenases of Mtb could be potential target antigens for vaccine development.

The greatest disadvantage of DNA vaccines is their relatively poor immunogenicity in humans, and although their safety has been proven in several human trials (Hobernik and Bros, 2018), they harbour a risk for stable integration of exogenous DNA or inflammatory and autoimmune reactions due to long-term expression of antigens (Hobernik and Bros, 2018). To overcome the poor immunogenicity of DNA vaccines in humans, several strategies – including enhanced delivery, inclusion of molecular adjuvants and improved vectors – are constantly being explored (Suschak et al., 2017). Next, we discuss how zebrafish can help to advance one of these strategies, the development of delivery methods for DNA vaccines.

### Zebrafish as a model for vaccine delivery

The limited success of DNA vaccines in clinical trials has been linked to the poor expression of vaccine-encoded antigens and insufficient antigen presentation, which, in turn, are at least partly caused by the inefficient delivery of the vaccine to the recipient cells. Nanomedicine is one possible solution for improving DNA delivery. In addition to vaccines, nanobiotics have been suggested to improve delivery of therapeutics against TB (Batalha et al., 2019).

Delivery vehicles investigated in zebrafish include single-walled carbon nanotubes (SWCNTs), nanoliposomes and biocompatible non-toxic approved materials, such as polymer-based nanoparticles [e.g. poly(lactic acid) (PLA) or poly(lactic-co-glycolic acid) (PLGA)] and other polymers (Zhang et al., 2020; Crecente-Campo et al., 2019; Ji et al., 2019; Guo et al., 2018; Lovmo et al., 2017; Resseguier et al., 2017; Ruyra et al., 2014). Studies in both adult and larval zebrafish have demonstrated that nanoparticles can cross the tissue epithelium and be taken up by antigen-presenting cells (Zhang et al., 2020; Resseguier et al., 2017) and other leukocytes (Lovmo et al., 2017). Crecente-Campo et al. (2019), in turn, have investigated the effect of particle size on phagocytosis and antigen presentation by macrophages. They found that smaller particles are more efficiently phagocytosed by macrophages (Crecente-Campo et al., 2019).

In larval zebrafish, lipopolysaccharide (LPS)- and polyinosinic:polycytidylic acid [poly(I:C)]-loaded nanoliposomes protected them from a sublethal challenge with *Aeromonas hydrophila* (Ji et al., 2019). Similarly, SWCNT-loaded recombinant proteins protected zebrafish against an *A. hydrophila* challenge, delivered both by bath immunization and by intraperitoneal injection (Guo et al., 2018). A similar study has been performed with nanoliposome-coated LPS and poly(I:C) against a spring viremia carp virus infection and *Pseudomonas aeruginosa* in adult zebrafish (Ruyra et al., 2014). The authors also discovered that lyophilised nanoliposomes provided comparable protection to the freshly prepared liposomes, further improving their usefulness (Ruyra, et al., 2014).

## Fish vaccines against mycobacterial infection

Besides zebrafish, several fish species have been used for modelling mycobacterial infections, as they are natural hosts to an aquatic mycobacterium. Armed with both innate and adaptive immunities, they also share key features of immune responses with humans (Rauta et al., 2012). Many of the studies have been designed with aquaculture in mind, but some information is also valid for human TB. However, vaccination and exposure strategies, such as balneation, that are commonly applied in aquacultural research are hardly applicable to mammals or humans. The most common models of a mycobacterial infection are the above-discussed zebrafish (Davis et al., 2002), medaka (Broussard and Ennis, 2007) and goldfish (Talaat et al., 1998), as these are aquarium sized and some unique genetic tools are available for each species. However, to our knowledge, medaka and goldfish have not been used for vaccination studies against mycobacterial species. Still, vaccination against mycobacteriosis has been studied in many other species important for commercial aquaculture, such as sea bass (*Dicentrarchus labrax*) (Ravid-Peretz et al., 2019; Ziklo et al., 2018), Japanese flounder (*Paralichthys olivaceus*) (Kato et al., 2010) and amberjack (*Seriola dumerili*) (Kato et al., 2011), and key results from vaccination experiments are displayed in Table 4. Notably, attenuated and killed mycobacteria (Ravid-Peretz et al., 2019; Ziklo et al., 2018), as well as BCG (Kato et al., 2011, 2010) and a DNA vaccine encoding Ag85A (Pasnik and Smith, 2005), have been shown to protect fish from mycobacteriosis. Studying the protective immune responses in these fish species could provide clues about the mammalian response to Mtb and help improve vaccine development efforts.

**Table 4.**
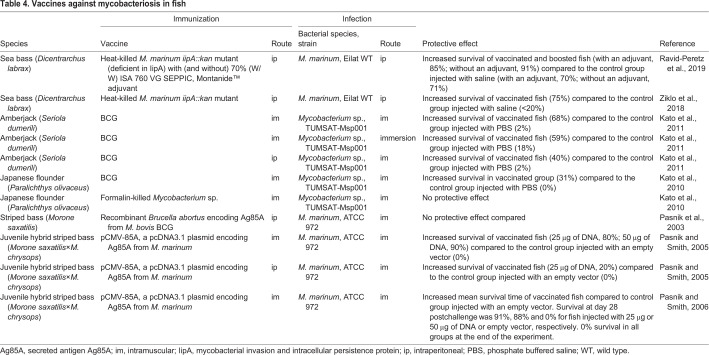
**Vaccines against mycobacteriosis in fish**

## Conclusions

As the role of the zebrafish in TB vaccine development begins to unfold, the relevance of the discoveries made in fish for humans remains to be determined. Despite the anatomical, genetic and physiological differences between zebrafish and humans, they appear to respond to the BCG vaccine and to antimicrobials that target mycobacteria in a similar way. These similarities, together with the novel insights into TB pathology gained from zebrafish, reflect the potential of this model for vaccine research. Compared to mammals, zebrafish are superior for large-scale screens, which allow the efficient discovery of protective antigens, antigen combinations and adjuvants to aid TB vaccine development. Furthermore, although not widely studied, fish also provide an attractive model for mucosal vaccine delivery. Mucosal administration through inhalation or oral delivery in humans does not require medical expertise or sterility, unlike invasive immunization methods, making it attractive for large-scale vaccination campaigns (Stylianou et al., 2018). Although vaccination routes in fish are typically intramuscular or intraperitoneal, immersion- or oral-based vaccination strategies could provide important information on the permeability of compounds to MALTs. Although there is evidence of the existence of the counterparts for the mammalian antigen-presenting mucosal cells (microfold cells) in Atlantic salmon (Fuglem et al., 2010), carp (Rombout et al., 1985) and zebrafish (Brugman, 2016), the function of these cells needs further characterization to establish the similarities and differences between mammals and fish and to validate these fish species as appropriate models of mammalian MALTs.

Although the availability of zebrafish-specific antibodies is continuously increasing, the persistent scarcity of immunological reagents still forces most of the early-stage immunogenicity studies to be performed in mice, a model that only partially recapitulates human TB and requires significantly more resources. For the same reason, the potential of zebrafish as a model to reveal the yet unknown protective mechanisms against TB cannot be fully harvested. While it is unlikely that zebrafish or other fish models will replace mammalian models in preclinical evaluation of novel vaccine candidates, they do provide a useful model for efficient and cost-effective early-phase screening of novel vaccine candidates and, in this way, minimize the time, costs and number of higher vertebrates needed on the long path to a vaccine.
